# Geneticand phenotypic characterization of a novel ST45-K43 carbapenem-resistant *Klebsiella pneumoniae* strain causing bloodstream infection: a potential clinical threat

**DOI:** 10.1128/spectrum.00305-24

**Published:** 2024-09-17

**Authors:** Xiaobing Chu, Xinmiao Jia, Peiyao Jia, Ying Zhu, Wei Yu, Xiaoyu Liu, Qiwen Yang

**Affiliations:** 1 Department of Clinical Laboratory, State Key Laboratory of Complex Severe and Rare Diseases, Peking Union Medical College Hospital, Chinese Academy of Medical Sciences and Peking Union Medical College, Beijing, China; 2 Graduate School, Peking Union Medical College, Chinese Academy of Medical Sciences, Beijing, China; 3 Center for bioinformatics, National Infrastructures for Translational Medicine, Institute of Clinical Medicine & Peking Union Medical College Hospital, Chinese Academy of Medical Sciences and Peking Union Medical College, Beijing, China; Beijing Institute of Genomics, Chinese Academy of Sciences, Beijing, China

**Keywords:** CRKP, ST45-K43, bloodstream infection, high transmission risk

## Abstract

*Klebsiella pneumoniae* is a common pathogen of nosocomial infection, which can cause pneumonia, urinary tract infection, cystitis, and bloodstream infections (BSIs). Here, we genetically characterize a novel carbapenem-resistant *K. pneumoniae* (CRKP) strain recovered from the blood of a 44-year-old male patient with severe acute necrotizing pancreatitis and septic shock in China. The strain is a ST45 *K. pneumoniae* with a novel serotype of K43, named 18SHX166. The susceptibility testing results showed that 18SHX166 was resistant to cephalosporin and carbapenems but still susceptible to ceftazidime-avibactam, quinolones, colistin, and amikacin. Genomic sequencing revealed that 18SHX166 contains three plasmids, namely pSHX166-Hv, pSHX166-KPC, and pSHX166-3. pSHX166-Hv harbored the *iucABCD* operon, encoding the siderophore of aerobactin. pSHX166-KPC harbored *bla*_KPC-2_ gene and possessed complete conjugative regions. The conjugation experiment verified pSHX166-KPC as a self-transmissible plasmid mediating the dissemination of antibiotic resistance, with a conjugation rate of 2.21 × 10^−5^. Additionally, the growth curve showed that 18SHX166 demonstrates a higher growth rate than the control strains. The characteristics of 18SHX166 indicate a potential high risk of clinical transmission.

**IMPORTANCE:**

ST45-K43 carbapenem-resistant *Klebsiella pneumoniae* isolate, 18SHX166, carries a carbapenem resistance plasmid and virulence plasmid. It has the characteristics of multidrug resistance, high transmissibility, and a fast growth rate, which could pose a threat to the control of antimicrobial resistance and clinical transmission, causing a severe challenge to public health.

## INTRODUCTION

*Klebsiella pneumoniae* is one of the most commonly isolated Gram-negative bacteria from bloodstream infections (BSIs) worldwide (1). Patients are often accompanied by multiple complications, poor prognosis, and high mortality rates (2). The presence of antibiotic-resistant *K. pneumoniae*, especially carbapenem-resistant strains, is linked to higher mortality rates (3). According to the surveillance antimicrobial resistance program in China, the isolation rate of *K. pneumoniae* from blood cultures was 18.8% in 2013, with an overall carbapenem resistance rate of 5.5% (4). The proportion of blood infections caused by a carbapenem-resistant *K. pneumoniae* (CRKP) has sharply increased, rising from 1.3% in 2009 to 26.7% in 2011 and reaching a peak of 34.3% in 2013, before fluctuating between 26.8% in 2018, 29.5% in 2020, and 26.7% in 2021 (5). Recent multicenter research in China shows that the sequence type ST11 (66.6%) is the main clone group isolated from bacteremic patients, followed by ST45 at 8.2% (6). ST45 is primarily linked to nosocomial transmission, with less common inter-hospital transmission (6).

In this study, we isolated a ST45 *K. pneumoniae* strain with a novel serotype of K43 from a patient with BSI. This strain simultaneously carried a carbapenem resistance plasmid and a virulence plasmid and also exhibited a high transmissible phenotype, which could pose a challenge to public health and the control of antimicrobial resistance.

## MATERIALS AND METHODS

### Bacterial strains

The *K. pneumoniae* isolate 18SHX166 was collected from the blood of a 44-year-old Chinese male patient with a blood infection in Chengdu, Sichuan, China on 25 March 2018 and was identified by Vitek MS MALDI-TOF (BioMérieux) system in Peking Union Medical College Hospital.

### Gene feature analysis

Genomic DNA extraction, sequencing, assembly, correction, and annotation are conducted according to our previous work (7). Antimicrobial resistance genes were identified using ResFinder 4.0 (8). Virulence genes were searched using the Virulence Factor Database (9). Conjugative transfer elements were identified using oriTfinder (10). Whole genome alignment was conducted using progressiveMauve (11). Pairwise sequence comparisons were also performed using BLAST. BRIG software was used for the generation of plasmid circular structure maps.

### Serotype and phylogenetic analysis

Sequence types were determined based on multilocus sequence typing (https://cge.cbs.dtu.dk/services/MLST/). It was also re-confirmed using SRST2 based on the Illumina reads (12). Serotype was analyzed using SerotypeFinder 2.0 based on the assembled contigs (13). A total of 15 ST45 *K. pneumoniae* strains with complete genome sequences were downloaded from NCBI Genbank database up to October 2023. The phylogenetic tree was constructed using FastTree based on the core-genome single nucleotide polymorphisms among the 15 downloaded strains and 18SHX166 (14), which were detected by MUMmer 3.23.

### Phenotypic characterization

The minimum inhibitory concentrations (MICs) were determined by broth microdilution method according to CLSI M100-S33 documents (15). *Escherichia coli* ATCC25922 and *Pseudomonas aeruginosa* ATCC27853 were used as quality controls.

To evaluate the virulence of 18SHX166, we used a mouse infection model in the study to compare the survival rates of the HvKP reference strain NTUH-K2044 (16) (high-virulence control strain), the classic *K. pneumoniae* strain QD110 (17) (low-virulence control strain), and 18SHX166.

For the mouse experiment, each mouse was intraperitoneally injected with 100 µL of bacterial suspension containing 10^7^ CFU. The survival status was observed every 12 hours for seven consecutive days. Each treatment group consisted of five mice.

Growth curve experiments were conducted on 18SHX166 using 0.5 McFarland suspensions prepared in LB medium, diluted 1:100, and incubated at 37°C for 24 hours with OD_600_ measurements taken every 10 minutes. We also selected the representative strains of ST23-K1 and ST65-K2 HvKPs, as well as ST11-K64 and ST11-K47 CR-HvKPs, with four strains of each type as controls. Biofilm formation capabilities were evaluated according to a previous report (18). Three technical replicates were performed for each strain. For the conjugation experiment, the 18SHX166 strain was used as the donor strain, and *E. coli* EC600 was used as the recipient strain. The experimental procedures were conducted as previously described (19). The antibiotic and the corresponding concentration used for each pair of conjugates are listed in Table S1. The transconjugants were further validated by PCR detection. According to the colony forming unit (CFU) count on the serial dilution plates containing corresponding antibiotics, the conjugation frequency was calculated as the ratio of transconjugants to recipients.

## RESULTS

### Clinical information

The clinical *K. pneumoniae* strain 18SHX166 was isolated from the blood culture of a 44-year-old male with severe acute necrotizing pancreatitis, infected pancreatic necrosis, and septic shock. The patient was admitted to hospital on 1 March 2018, due to an infection of other abdominal organs caused by *K. pneumoniae*.

After the onset of BSI, the patient was immediately admitted to the intensive care unit and treated with a combination of cefoperazone-sulbactam, ceftriaxone, ciprofloxacin, tigecycline, meropenem, linezolid, and daptomycin. The strain 18SHX166 was isolated from blood culture on 25 March 2018, and no *K. pneumoniae* was isolated from other sites outside the patient's blood.

### Sequence typing and phylogenetic analysis

Sequence type and serotype analysis showed that 18SHX166 belongs to ST45 and K43. In order to study the evolutionary relationship between 18SHX166 and other ST45 strains, we downloaded the whole genome sequences of *K. pneumoniae* strains from NCBI and obtained 15 ST45 strains. Analysis of the serotypes of these ST45 strains revealed that only 18SHX166 was of K43 type. From the result of phylogenetic analysis, we can see that 18SHX166, as a separate branch, is more close to the K23 cluster (Fig. S1).

### Genomic characteristics and antimicrobial resistance/virulence genotype analysis

**Fig 1:**
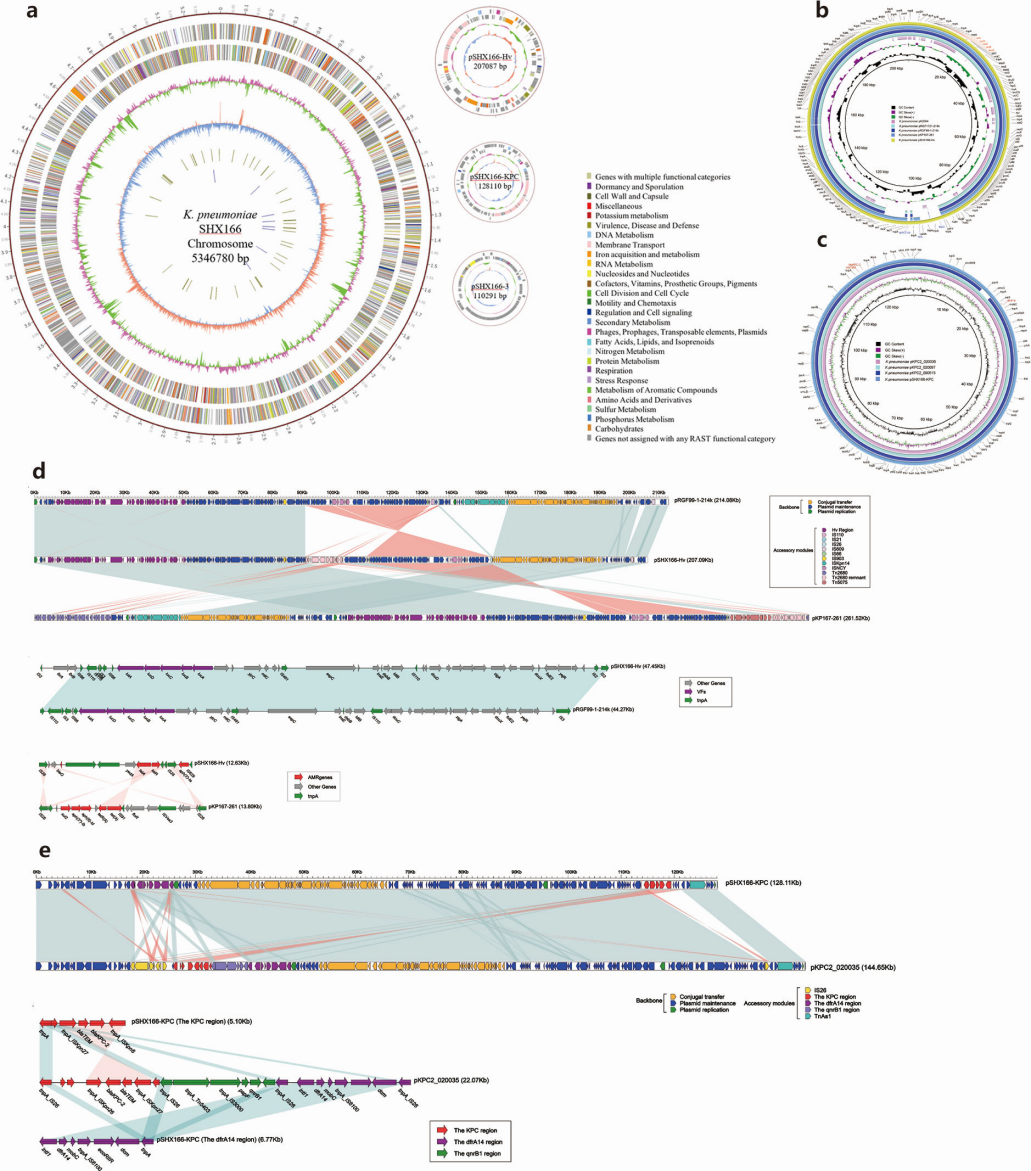
The genomic characteristics of 18SHX166 (chromosome and three plasmids). (**a**) The complete genome of the clinical isolate 18SHX166, including a 5.3 Mb chromosome, a 207 kb plasmid (pSHX166-Hv), a 128 kb plasmid (pSHX166-KPC), and a 110 kb plasmid (pSHX166-3). (**b**) Genetic structure of pSHX166-Hv. (**c**) Genetic structure of pSHX166-KPC. (**d**) Structure of pSHX166-Hv and related transposon. (**E**) Structure of pSHX166-KPC and related transposon.

To reveal the genetic basis of the multidrug-resistant and virulent phenotype, we obtained the complete genome of the clinical isolate 18SHX166 including a 5.3 Mb chromosome, a 207 kb plasmid (pSHX166-Hv), a 128 kb plasmid (pSHX166-KPC), and a 110 kb plasmid (pSHX166-3) (Fig. 1a). Bioinformatics analysis provided the general information of the chromosome genome, including GC% content (57.48%), predicted protein-coding genes (5,194), average gene length (914 bp), and coding region (88.81%). The three plasmids possess a shorter average gene length, lower ratio of coding regions, and lower GC% content (Table 1).

**TABLE 1:** The genomic characteristics and antimicrobial resistance genotype analysis of the sequences of chromosomes and plasmids of 18SHX166

Name^*a*^	Genome size (bp)	GC content	Coding genes	Average gene size (bp)	Coding region (bp)	tRNA	rRNA	Virulence genes	Drug resistance genes	Plasmid type
SHX166_chr	5,346,780	57.48%	5,194	914	4,748,439 (88.81%)	87	25	*fyuA, irp, iutA, mrkABCDFHIJ, wzi, ybtAEPQSTUX*	*fosA7, oqxA, oqxB, bla*_SHV26_	/
pSHX166-Hv	207,087	51.34%	228	744	169,614 (81.90%)	/	/	*iucABCD, iutA*	*aph(3′)-Ia, bleO, tet(A*)	IncFIB(K)
pSHX166-KPC	128,110	52.12%	157	656	103,053 (80.44%)	/	/	/	*dfrA14, bla_TEM1B_, bla*_KPC-2_	IncFII(K)/IncR/IncFII(Yp)
pSHX166-3	110,291	49.19%	117	809	94,680 (85.85%)	2	/	/	/	/

^*a*^ Sequences of chromosomes end in “chr”; sequences of plasmids begin with “p”.

^*b*^ “/” indicates no relevant gene or plasmid.

There are four antimicrobial resistance genes in the chromosome: *oqxA* and *oqxB* (a quinolone and amphenicol efflux pump), the fosfomycin-modifying enzyme-encoding gene *fosA7*, and β-lactam resistance gene *bla*_SHV_. There are three antimicrobial resistance genes in the plasmid pSHX166-Hv: aminoglycoside resistance gene [*aph(3′)-Ia*], glycopeptide resistance gene (*bleO*), and tetracycline resistance [*tet(A*)]. Three antimicrobial resistance genes are located on the plasmid pSHX166-KPC, including carbapenemase resistance gene *bla*_KPC-2_, β-lactam resistance gene *bla*_TEM_, and trimethoprim resistance gene *dfrA14* (Table 1). The plasmid pSHX166-3 does not carry any antimicrobial resistance genes (Fig. S2).

The analysis of virulence-related genes indicated that the chromosome of strain 18SHX166 harbors five types of virulence-related genes, encompassing those encoding aerobic actin synthesis and transport protein (*iutA*), Yersinia receptor (f*yuA*), pili cluster (*mrkABCDFHIJ*), capsule assembly protein (*wzi*) in the Wzi family, and Yersinia abactin gene (*ybtAEPQSTUX*) (Table 1).

### Characterization of plasmids pSHX166-Hv and pSHX166-KPC

Genome analysis indicated that 18SHX166 contains one virulence plasmid, pSHX166-Hv, belonging to IncFIB(K) group, which carried five virulence genes (*iucABCD*, *iutA*) and three antibiotic resistance genes [*aph(3′)-Ia*, *bleO*, and *tet(A*)]. Comparative analysis shows that pSHX166-Hv shares high similarity with three *K. pneumoniae* plasmids (pRGF1721-214k, pRGF99-1-214k, and pKP167-261), with 90–95% coverage and 99.99% identity (Fig. 1b). We also compared the pSHX166-HV and pK2044, and the results indicated that there is a large difference in structure and sequence between them (Fig. 1b; Fig. S3). Additionally, the bleomycin resistance gene *bleO* on pSHX166-Hv is situated in the Tn*2680* remnant region (Fig. 1d), a fragment of the composite transposon Tn*2680* that plays an important role in bacterial adaptation to environmental changes and the dissemination of antibiotic resistance genes.

The plasmids pSHX166-KPC, which contains the carbapenemase resistance gene *bla*_KPC-2_, belongs to the IncFII(K)/IncR/IncFII(Yp) group and carries three drug resistance genes, *dfrA14*, *bla*_TEM_, and *bla*_KPC-2_. Sequence analysis reveals a 99% identity and 99% coverage with three *K. pneumoniae* plasmids (pKPC2_020035, pKPC2_020097, and pKPC2_090515 ) (Fig. 1c). *bla*_KPC-2_ is typically located within the transposon Tn*6296*, so we compared the gene environment of *bla*_KPC-2_ in pSHX166-KPC and Tn*6296*. The KPC region in pSHX166-KPC only retains the part from ISKpn6 transposase to IS*26* transposase in Tn*6296* and lacks genes from *korC* to IS*26* transposase compared to Tn*6296* (Fig. S4). Additionally, due to the high similarity in accessory regions between pSHX166-KPC and pKPC2_020035, a further linear genomic comparison was conducted between them (Fig. 1e). In addition to harboring the *bla*_KPC-2_ gene, pSHX166-KPC contains *TraX*, *TraG*, *TraF*, *TraB*, *TraH*, *TraQ*, *TraX*, *TraN*, *TraL*, *TraU*, *TraW*, *TraV*, *TraA*, *TraM*, *TraK*, *TraE* , and *TrbC* belonging to T4SS; *TraI* belonging to the *oriT* region and relaxase; and *Trad* belonging to T4CP. These genes are all associated with conjugative system, indicating that it may be a self-transmissible plasmid mediating the dissemination of antibiotic resistance.

### Phenotype and genotype of 18SHX166

The antimicrobial susceptibility test results revealed that the clinical isolate *K. pneumoniae* 18SHX166 is resistance to multiple antimicrobial agents, including Cefepime, Aztreonam, Piperacillin-tazobactam, Ceftazidime, Meropenem, Ceftriaxone, Imipenem, Ceftolozone-tazobactam, Ertapenem, and Colistin, but remains susceptible to amikacin, Ceftazidime-avibactam, Ciprofloxacin, Levofloxacin, and Imipenem-relebactam (Table 2).

**TABLE 2:** The minimum inhibitory concentrations (MICs) for 18SHX166 to 16 antimicrobial agents^*a*^

Antimicrobial agent	MIC (mg/L)	Clinical breakpoint	Interpretation
Colistin	≤1	I ≤ 2 R ≥ 4	I
Piperacillin-tazobactam	>64/4	S ≤ 8/4 R ≥ 32/4	R
Amikacin	≤4	S ≤ 16 R ≥ 64	S
Aztreonam	>16	S ≤ 4 R ≥ 16	R
Ceftazidime	8	S ≤ 4 R ≥ 16	R
Ceftazidime-avibactam	0.25	S ≤ 8/4 R ≥ 16/4	S
Meropenem	>8	S ≤ 1 R ≥ 4	R
Imipenem	8	S ≤ 1 R ≥ 4	R
Ertapenem	＞4	S ≤ 0.5 R ≥ 2	R
Ciprofloxacin	≤0.25	S ≤ 0.25 R ≥ 1	S
Levofloxacin	≤0.5	S ≤ 0.5 R ≥ 2	S
Cefepime	>16	S ≤ 2 R ≥ 16	R
Imipenem-relebactam	0.25	S ≤ 1/4 R ≥ 4/4	S
Ceftolozone-tazobactam	8	S ≤ 2/4 R ≥ 8/4	R
Cefoxitin	8	S ≤ 8 R ≥ 32	S
Ceftriaxone	>8	S ≤ 1 R ≥ 4	R

^*a*^ MIC, minimal inhibitory concentration; S, susceptible; I, intermediate; R, resistant.

**Fig 2:**
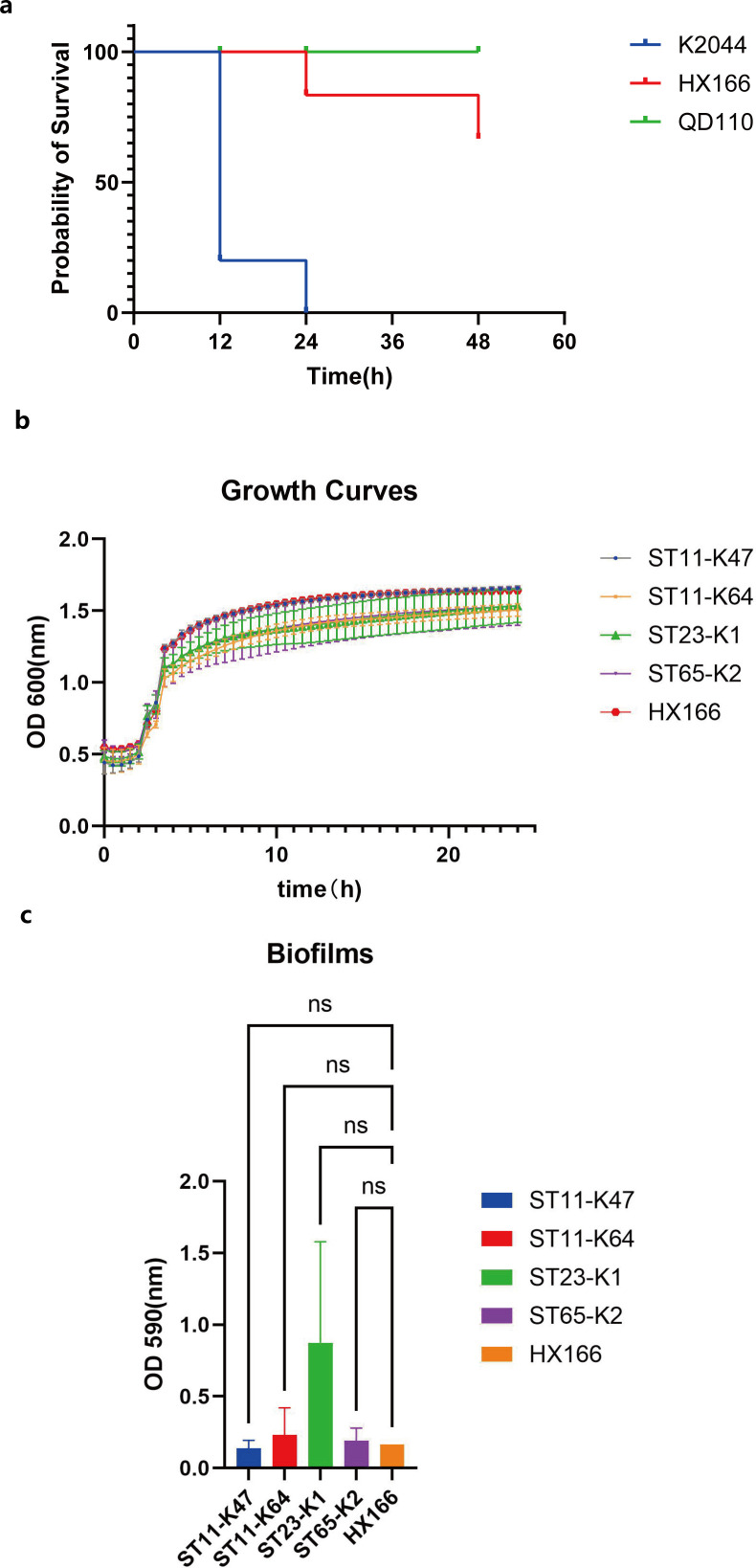
The phenotypic characterization of 18SHX166. (**a**) Survival curves that define the virulence phenotype of 18SHX166 in mouse survival assay. (**b**) The growth curves of 18SHX166 were compared with 16 strains of *K. pneumoniae* of four different sequence types. (**c**) The biofilm formation ability of 18SHX166 compared with 16 strains of *K. pneumoniae* of four different sequence types (ns means *P* > 0.05).

After 7 days of infection, the 18SHX166 strain demonstrated a low-virulence phenotype in a mouse model, with an 80% survival rate, which was equivalent to that of QD110 (100% survival rate). In contrast, the NYUH-K2044 strain maintained a 100% mortality rate throughout the entire experiment. Compared to NTUH-K2044 and QD110, 18SHX166 demonstrated a lower virulence phenotype in the mouse survival assay (Fig. 2a).

The conjugation experiment showed that pSHX166-KPC plasmid could be transferred to *E. coli* EC600 via conjugation, with an efficiency of 2.21E-05, indicating that it is a self-transmissible plasmid mediating the dissemination of antibiotic resistance. The growth curve indicates that 18SHX166 has a faster growth rate, but there is no statistically significant difference in growth rate and biofilm formation ability compared to the other 16 strains (Fig. 2b and c).

## DISCUSSION

In this study, we conducted a genetic and phenotypic analysis on a novel ST45-K23 carbapenem-resistant strain of *K. pneumoniae*, designated as 18SHX166, which exhibited a multidrug-resistant phenotype, high transmissibility, and a fast growth rate.

Previous research in China has shown that ST11 is the predominant clone among bacteremic patients, followed by ST45 (6). This suggests an increasing prevalence of ST45 *K. pneumoniae* in China, which could lead to nosocomial infections. It is well known that polluted environments in hospitals play an important role in the spread of bacteria, and studies have shown that the spread of ST45 extended-spectrum β-lactamase producing *K. pneumoniae* in neonatal intensive care unit is related to environmental pollution (20). ST45 *K. pneumoniae* has been observed in Portugal and has even been associated with hospital outbreaks caused by multidrug-resistant, KPC-3- and MCR-1-producing strains (21). The conjugation experiment showed that pSHX166-KPC plasmid could be transferred to *E. coli* EC600 via conjugation, with an efficiency of 2.21E-05, indicating its potential to disseminate antibiotic resistance through self-transmission. The growth curve shows that 18SHX166 exhibits a faster growth rate and demonstrates a certain growth advantage. Despite possessing the *iutA*, *fyuA*, *mrkABCDFHIJ*, *wzi*, *ybtAEPQSTUX*, and *iucABCD* genes, 18SHX166 *K. pneumoniae* lacks traditional hypervirulence gene markers, such as *rmpA*, *rmpA2*, *iroB*, and *peg344* (22). This may contribute to its low-virulence phenotype.

In conclusion, this study identified a CRKP strain 18SHX166 causing BSIs, which is a strain of ST45 *K. pneumoniae* with a novel K type (K43) that carries a carbapenem resistance plasmid and a virulence plasmid. 18SHX166 exhibited multidrug resistance, high transmissibility, and a fast growth rate. The spread of *K. pneumoniae* ST45 is associated with environmental pollution and has the risk of nosocomial infection outbreak. The discovery of the ST45-K43 CRKP isolate could pose a threat to the control of antimicrobial resistance and could cause a severe challenge to public health.

## Data Availability

The genome data of K. pneumoniae 18SHX166 were deposited in NCBI under BioProject ID PRJNA692086 with accession number CP139926-CP139929. Other NCBI genomic data sources used in the article: K. pneumoniae pRGF1721-214k with accession number CP075277. K. pneumoniae pRGF99-1-214k with accession number CP075553. K. pneumoniae pKP167-261 with accession number CP098759. K. pneumoniae pKPC2_020035 with accession number CP045990. K. pneumoniae pKPC2_020097 with accession number CP043349. K. pneumoniae pKPC2_090515 with accession number CP073288. *K. pneumoniae* plasmid p628-KPC with accession number NC_032103.1.

## SUPPLEMENTAL MATERIAL

Fig. S1 spectrum.00305-24-s0001.tiff Phylogenic tree of 18SHX166 with 15 other strains of ST45 *K. pneumoniae* in the NCBI database.

Fig. S2 spectrum.00305-24-s0002.tif Genetic structure of pSHX166-3.

Fig. S3 spectrum.00305-24-s0003.tiff Sequence and structure comparison of pSHX166-Hv and pK2044.

Fig. S4 spectrum.00305-24-s0004.tif The genetic environment of blaKPC in pSHX166-KPC and the structural comparison with Tn*6296*.

Supplemental material spectrum.00305-24-s0005.docx Legends.

Table S1 spectrum.00305-24-s0006.docx Antibiotics and the corresponding concentrations used for conjugation pair and the CFU counts.

ASM does not own the copyrights to Supplemental Material that may be linked to, or accessed through, an article. The authors have granted ASM a non-exclusive, world-wide license to publish the Supplemental Material files. Please contact the corresponding author directly for reuse.
