# Supplementary figures and images for: Geneticand phenotypic characterization of a novel ST45-K43 carbapenem-resistant Klebsiella pneumoniae strain causing bloodstream infection: a potential clinical threat
Source: Microbiol Spectr. 2024 Sep 17;12(11):e00305-24. doi: 10.1128/spectrum.00305-24 (PMC11537024; doi:10.1128/spectrum.00305-24)

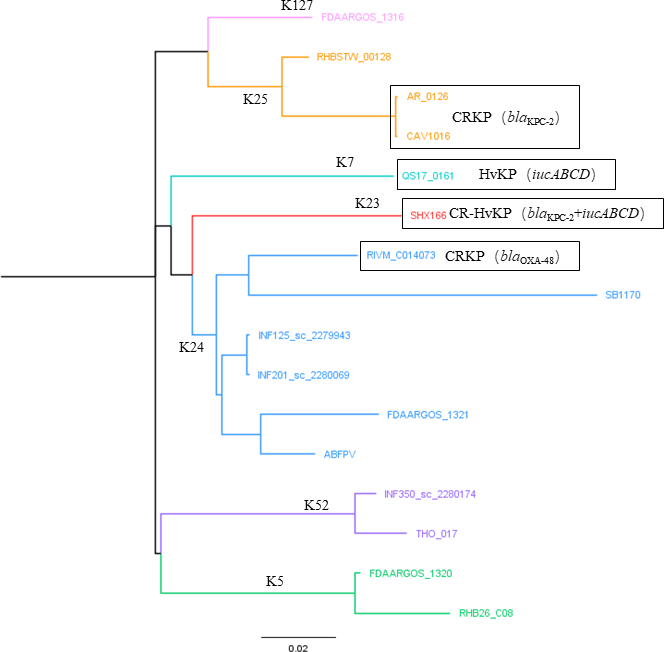

Supplement: Fig. S1 — Phylogenic tree of 18SHX166 with 15 other strains of ST45 K. pneumoniae in the NCBI database. [file spectrum.00305-24-s0001.tiff]

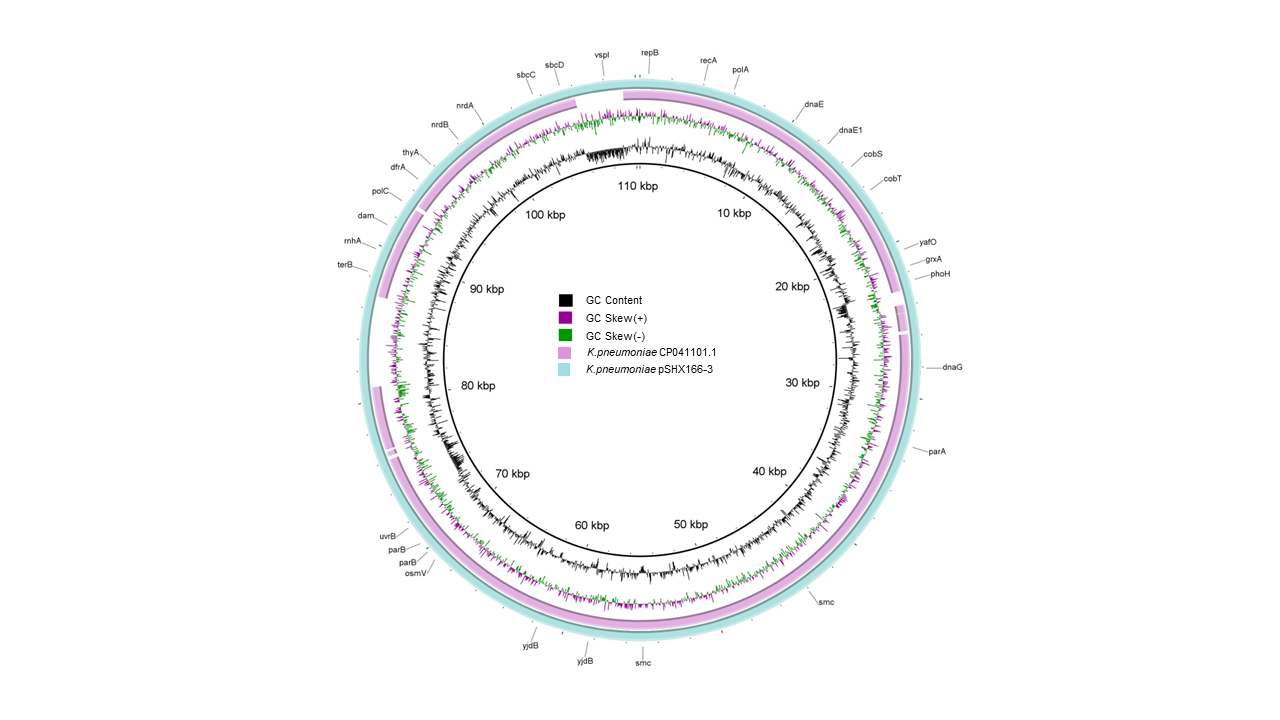

Supplement: Fig. S2 — Genetic structure of pSHX166-3. [file spectrum.00305-24-s0002.tif]

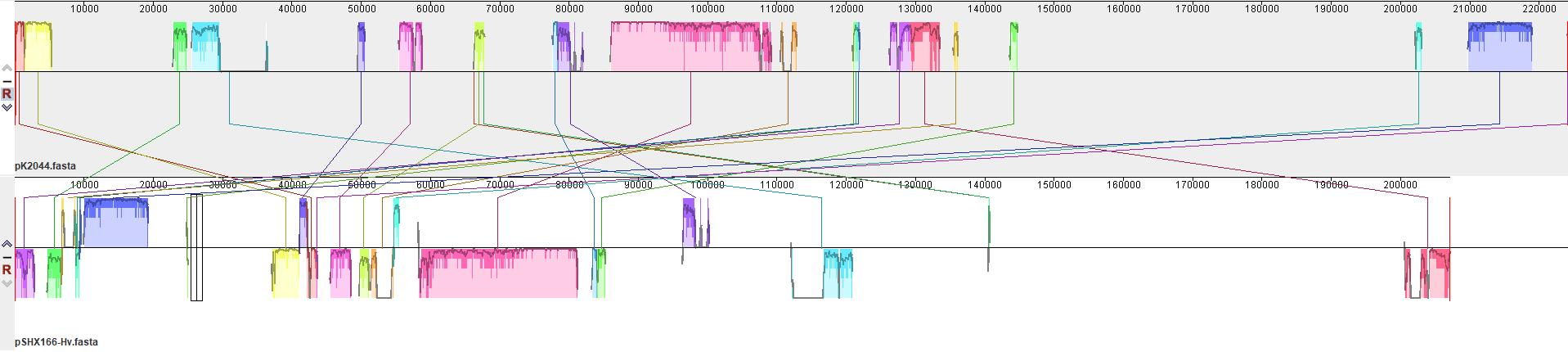

Supplement: Fig. S3 — Sequence and structure comparison of pSHX166-Hv and pK2044. [file spectrum.00305-24-s0003.tiff]

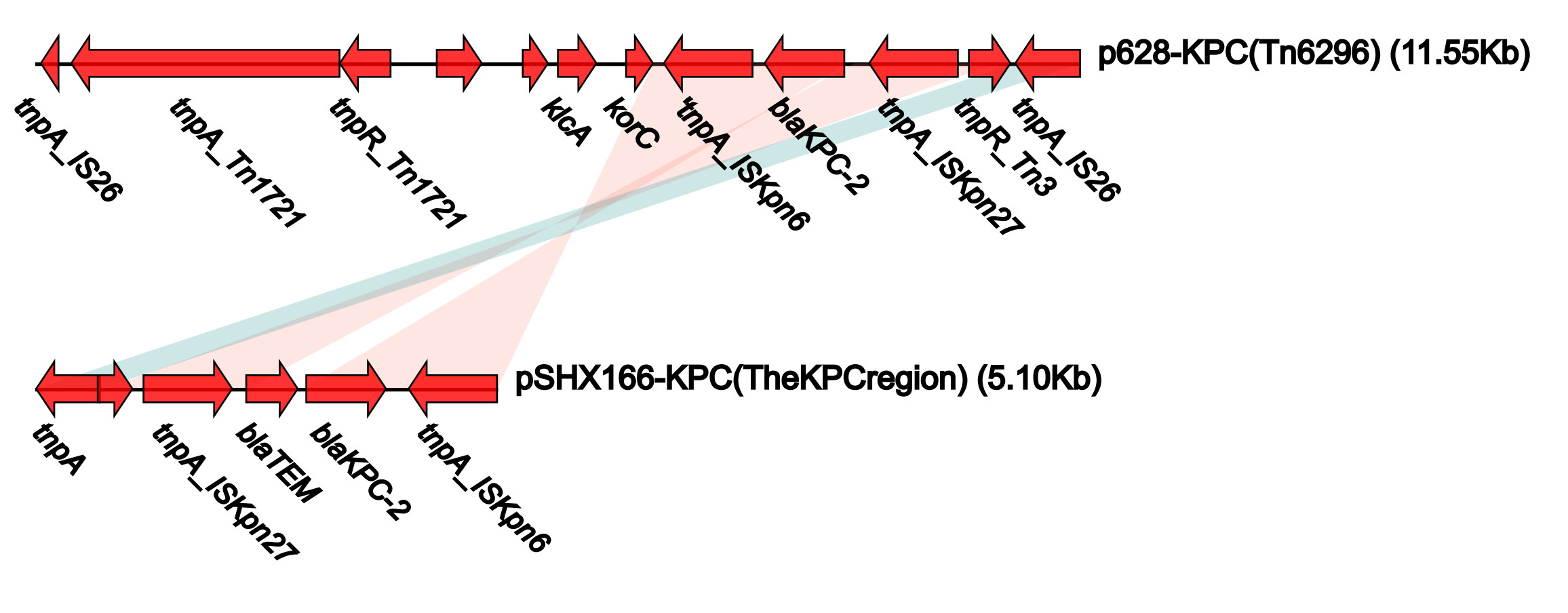

Supplement: Fig. S4 — The genetic environment of blaKPC in pSHX166-KPC and the structural comparison with Tn6296. [file spectrum.00305-24-s0004.tif]
