# Supplementary material for: Geneticand phenotypic characterization of a novel ST45-K43 carbapenem-resistant Klebsiella pneumoniae strain causing bloodstream infection: a potential clinical threat
Source: Microbiol Spectr. 2024 Sep 17;12(11):e00305-24. doi: 10.1128/spectrum.00305-24 (PMC11537024; doi:10.1128/spectrum.00305-24)
Supplement: Supplemental material — Legends. [file spectrum.00305-24-s0005.docx]

Fig. S1 Phylogenic tree of 18SHX166 with 15 other strains of ST45 *K. pneumoniae* in the NCBI database.

Fig. S2 Genetic structure of pSHX166-3.

Fig. S3 Sequence and structure comparison of pSHX166-Hv and pK2044.

Fig. S4 The genetic environment of *bla*_KPC_ in pSHX166-KPC and the structural comparison with Tn6296.
