# Supplementary material for: Geneticand phenotypic characterization of a novel ST45-K43 carbapenem-resistant Klebsiella pneumoniae strain causing bloodstream infection: a potential clinical threat
Source: Microbiol Spectr. 2024 Sep 17;12(11):e00305-24. doi: 10.1128/spectrum.00305-24 (PMC11537024; doi:10.1128/spectrum.00305-24)
Supplement: Table S1 — Antibiotics and the corresponding concentrations used for conjugation pair and the CFU counts. [file spectrum.00305-24-s0006.docx]

Table S 1 Antibiotics and the corresponding concentrations used for conjugation pairand the colony-forming unit (CFU) counts.

| Donor | Recipient | Conjugates | Antibiotic and antibiotic concentration |
| --- | --- | --- | --- |
| SHX166 | EC600 |  | 200ug/ml Levofloxacin+1μg/ml meropenem |
|  | 264000000 | 5860 |  |
